# Performance analysis and optimization of inverted inorganic CsGeI_3_ perovskite cells with carbon/copper charge transport materials using SCAPS-1D

**DOI:** 10.1098/rsos.221127

**Published:** 2023-03-15

**Authors:** Waqar Ahmad, Muhammad Noman, Shayan Tariq Jan, Adnan Daud Khan

**Affiliations:** ^1^ U.S.-Pakistan Centre for Advanced Studies in Energy, University of Engineering and Technology, Peshawar 25000, Pakistan; ^2^ Department of Energy Engineering Technology, University of Technology, Nowshera 24100, Pakistan

**Keywords:** perovskite solar cells, CsGei_3_, inorganic, charge transport layers, SCAPS-1D

## Abstract

Organic–inorganic perovskite solar cells (PSCs) have achieved the power conversion efficiencies (PCEs) of more than 25%. However, the organic compound in the material is causing structural degradation of the PSC owing to heat (thermal instability), humidity and moisture. This has led to the exploration of only inorganic perovskite materials. Inorganic PSCs such as caesium have seen a breakthrough by achieving highly stable PSC with PCE exceeding 15%. In this work, the inorganic non-toxic PSC of caesium germanium tri-iodide (CsGeI_3_) is numerically modelled in SCAPS-1D with two carbon-based electron transport layers (ETLs) and two copper-based hole transport layers (HTLs). This study introduces in-depth numerical modelling and analysis of CsGeI_3_ through continuity and Poisson equations. Cu HTLs are selected to increase the electric conductivity of the cell, while carbon-based ETL is used to increase the thermal conductivity of the PSC. A total of four unique PSC structures are designed and presented. A systematic approach is adopted to obtain the optimized PSC design parameters for maximum performance. From the optimized results, it is observed that the C_60_/CsGeI_3_/CuSCN structure is the highest performance PSC, with open-circuit voltage (*V*_oc_) of 1.0169 V, short-circuit current density (*J*_sc_) of 19.653 mA cm^−2^, fill factor of 88.13% and the PCE of 17.61%. Moreover, the effect of quantum efficiency, electric field, interface recombination, interface defects, layer thickness, defect density, doping concentration, working temperature and reflection coating on the cell performance are studied in detail.

## 1. Introduction

Perovskite materials are named after the crystal shape of the mineral CaTiO_3_. They have crystal structure of ABX_3_, where A represents the organic/inorganic cation, B represents the metal cation and X is the halide anion [1]. Perovskite solar cells (PSCs) are organic/inorganic metal halide photovoltaic cells. Recent advancement in the technology has enabled the fabrication of perovskite-based photovoltaics (PV) module, making them an alternative to traditional silicon-based PV modules. However, this technology still has a lot of space for improving their stability and performance.

Over the past few years, lead-based PSCs have brought a revolution in the PV industry with achieving power conversion efficiency (PCE) of more than 25% [2]. Although high efficiencies have been achieved, the long-term stability of the PSC owing to its organic component and the toxicity of the lead (Pb) still remains a significant concern of worry [3]. The organic part in the perovskite material quickly decomposes on exposure to moisture and heat. This causes the accelerated degradation of the PSC which leads to Pb exposure into the surrounding environment. To solve this issue, there is a need for an alternative potential material to replace the lead and organic compound in the perovskite material.

Inorganic materials like caesium (Cs) have been used in PSC absorbers to remove the organic component, reducing its decomposition rate. Cs-based PSCs have been found to be stable up to 150°C temperature, maintaining its efficiency and structure for longer duration. Using Cs instead of organic component reduces the band gap of the perovskite material, which in turn increases the absorption of the PSC. The increased absorption can be taken advantage of by finding high-performing Cs-PSC structures.

To remove the toxic factor, germanium (Ge) has emerged as an alternative for Pb in perovskite materials. Ge is from the same group of Pb and has similar photovoltaic properties. Recent theoretical studies of Ge-based PSC have shown a light conversion efficiency above 15% [4]. The Ge-PSC not only gives the advantage of being completely toxic free but it has also been found by density functional theory calculations to be potentially more stable than Sn-PSC [5].

Understanding the mechanics of how PV cells work is critical to improving the performance of Cs-Ge-based PSCs. The use of software simulation and numerical modelling is critical in understanding how the device works. A systematic approach through simulation tools can be adopted to optimize the cells and achieve higher PCE [6].

The basic PSC structure has three main materials, i.e. electron transport material (ETM), absorber and hole transport material (HTM). When the light strikes the absorber material, electron and hole pairs are created. The ETM is n-type material, used to separate the electrons from the absorber and block the holes from crossing into the cathode. While the HTM is p-type material used to separate the holes and block the electrons from the anode [7]. Selecting the right type of materials as a charge transport material (CTM) not only increases the PSC's PCE, but also its stability.

Studies have shown that using carbon-based charge transport layers (CTLs) in lead- and tin-based PSC structures increases the stability of the cell. This is because carbon is an excellent thermal conductor. They have extensively been used with Pb- and Sn-based PSC especially as the electron transport layer (ETL) in the structure. Using carbon as ETL also refines the shunt and series resistances owing to its conductive nature [8]. Another advantage of carbon is that it is excessively present in nature and easily accessible.

Copper as the hole transport layer (HTL) has also been presented to be a good option as the HTL in the Pb and Sn PSC structure, as it improves the efficiency of the cell owing to its superior electrical conductivity and high carrier mobility [9].

Multiple research works have found that using different combinations of ETL and HTL with a specific perovskite material produces varying results [10–12]. Identifying the best CTL combination for each perovskite material for improved performance is very vital. The same CTL combination does not produce the same results when used with other perovskite material absorbers. This is because each unique structure combination produces a separate energy band alignment, band gap, electric field and conductive potential, which influence the working mechanism of the PSC.

In this study, inverted inorganic CsGeI_3_-based PSCs are modelled with two carbon (C_60_ and PCBM)-based ETL and two copper (CuSCN and CuSbS_2_)-based HTL in SCAPS-1D. Cs-based Ge-PSC (CsGeI_3_) is not only a non-toxic inorganic material but also has band gap (1.65 eV) nearer to MAPbI_3_ (1.55 eV) than MA-based Ge-PSC (MAGeI_3_; 1.9 eV). The smaller band gap increases the absorption of the perovskite material. Copper is used to increase the PSC electric conductivity while carbon is used to increase the thermal conductivity. To achieve a stable and efficient CsGe-PSC, detailed simulations are carried out. A systematic approach is adopted to achieve optimized cell design parameters for enhanced performance of the different PSC structures. The effect of quantum efficiency, electric field, interface recombination, interface defects, layer thickness, defect density, doping concentration, working temperature and reflection coating on the cell performance are studied in detail.

## 2. Materials and methods

### 2.1. Software—SCAPS-1D

SCAPS-1D is a simulation tool developed by the University of Ghent's Department of Electronics and Information Systems, Belgium. Different cells structures can be designed using this software. Seven different layers can be added to the structure. As the cell structure is defined, the following differential equations are used to determine different parameters of the cell and its output performance:

Poisson equation,

2.1 $$\frac{\partial^{2}\varphi }{\partial^{2}x}=-\frac{\partial E}{\partial x}=-\frac{\rho }{\varepsilon_{s}}=-\frac{q}{\varepsilon_{s}}[\;p-n+N_{D}(x)-N_{A}(x)\pm N_{\mathrm{def}}(x)].$$

Transport equation,

2.2 $$J_{n,p}=nq\mu_{n}E+qD_{n}\frac{\partial n}{\partial x}+pq\mu_{p}E+qD_{p}\frac{\partial p}{\partial x}.$$

Diffusion length,

2.3 $$L_{n,p}=\sqrt{D_{n,p}\tau_{n,p}}.$$

Diffusivity,

2.4 $$D_{n,p}=\left[\left(\frac{K_{B}T}{q}\right)\mu_{n,p}\right].$$

Continuity equation,

2.5 $$\frac{\partial n,p}{\partial t}=\frac{1}{q}\frac{\partial J_{n}}{\partial x}+(G_{n}-R_{n})+\frac{1}{q}\frac{\partial J_{p}}{\partial x}+(G_{p}-R_{p}).$$

Open-circuit voltage,

2.6 $$V_{OC}=\frac{nK_{B}T}{q}\left[\ln\left(\frac{I_{L}}{I_{O}}+1\right)\right].$$

Carrier lifetime,

2.7 $$\mathrm{and}\;\tau =\;\frac{1}{\sigma *\;Nt*\;Vth}.$$

φ represents cell's electrostatic potential, *E* represents electric field, ρ and *q* represent elementary charge, ε is material's permittivity, and free electron/hole densities are represented by *n*.

The doping densities are represented by *N*_A_ for acceptor and *N*_D_ for donor. The e^−^/h^+^ mobility is represented by $\mu_{n,p}\;$, $\tau_{n,p}$ is the electron/hole lifetime, and ∂n,p/∂x represents charge carriers concentration gradient. Optical generation rate is represented by *G*_n,p_, recombination rate is represented by *R*_n,p_, thermal voltage is represented by $K_{B}T/q$, *I*_L_ is for the current generated by light, and *I*_o_ is for the saturation current.

In the presented work, all the simulations are carried out under standard test conditions (STCs) of 1.5 G air mass, 1000 W m^−2^ irradiance and 300 K temperature were used.

### 2.2. Device structure

The most commonly used structure for PSC has five basic thin film layers [9]. The first material used is of negative electrode (cathode). Its function is to collect the negative ions (electrons). The second layer is the ETM which is on top of the electrode. Its main function is to draw out the electrons from the absorber layer. Then comes the perovskite material which is used as the absorber layer in the cell. This layer when exposed to light produces the charge carriers. On the top of the perovskite layer is the HTM, the function of which is to separate the holes from the perovskite layer. The last and top most layer is of the positive electrode (anode) which collects the holes [13].

**Figure 1.**
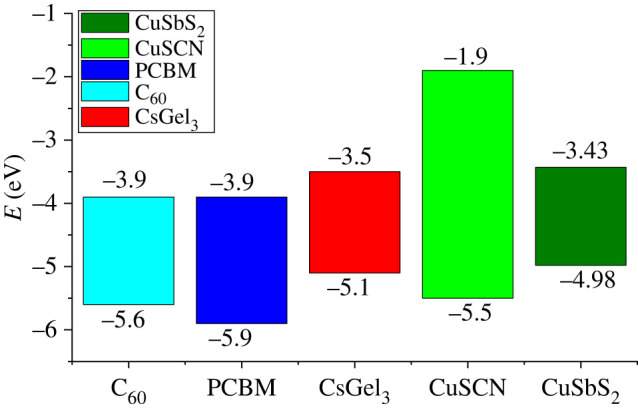
Energy states of PSC layers.

Caesium germanium tri-iodide (CsGeI_3_) is used as absorber material of perovskite in this study. The Cs inorganic nature and Ge non-toxic nature make it an ideal candidate to be used in PSC. It has band gap near to MAPbI_3_ and smaller than MAGeI_3_ perovskite material. Carbon-based ETMs (C_60_ and PCBM) along with copper-based HTMs (CuSCN and CuSbS_2_) are used as CTMs to increase the cell's thermal and electrical conductivity for higher performance.

To achieve maximum cell performance, the ideal energy states diagram is shown in figure 1.

**Figure 2.**
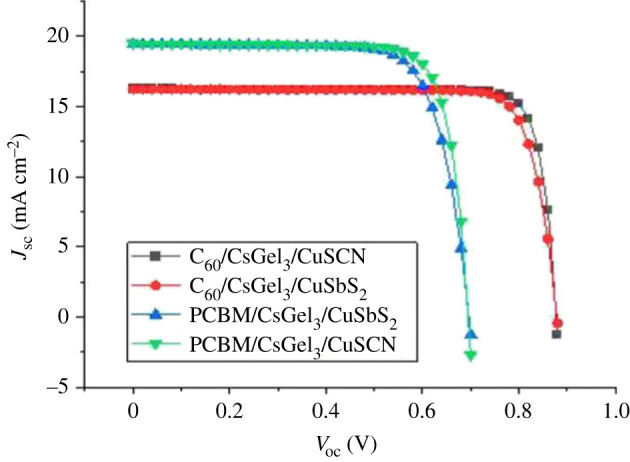
IV characteristics of simulated structures.

### 2.3. Device modelling

SCAPS-1D software is used to numerically model all the structures (cathode/ETM/CsGeI_3_/HTM/anode) layer by layer. In table 1, the design parameters of all the materials are mentioned, which have been obtained from a detailed study of different theoretical and experimental published research papers [14–16]. The work function for cathode and anode were taken as 4.4 and 5.1 eV, respectively.

**Table 1.** Simulation parameters list for PSC [14–17].

parameters	C_60_	PCBM	CsGeI_3_	CuSCN	CuSbS_2_
thickness (nm)	100	100	500	200	150
band gap (eV)	1.7	2.0	1.6	3.4	1.58
electron affinity (eV)	3.9	3.9	3.5	1.9	4.2
dielectric permittivity	4.2	3.9	18	9	14.6
conduction band effective density of state (cm^−3^)	8 × 10^19^	2.5 × 10^21^	1 × 10^18^	2.5 × 10^18^	2 × 10^18^
valance band effective density of state (cm^−3^)	8 × 10^19^	2.5 × 10^21^	1 × 10^18^	1.8 × 10^19^	1 × 10^18^
electron thermal velocity (cm s^−1^)	1 × 10^7^	1 × 10^7^	1 × 10^7^	1 × 10^7^	1 × 10^7^
hole thermal velocity (cm s^−1^)	1 × 10^7^	1 × 10^7^	1 × 10^7^	1 × 10^7^	1 × 10^7^
electron mobility (cm^2^ (V s)^−1^)	1 × 10^−2^	2 × 10^−1^	20	2 × 10^−4^	4.9 × 10^1^
hole mobility (cm^2^ (V s)^−1^)	3.9 × 10^−3^	2 × 10^−1^	20	1 × 10^−2^	4.9 × 10^1^
donor density *N*_D_ (cm^−3^)	2.6 × 10^18^	2.93 × 10^17^	—	—	—
acceptor density *N*_A_ (cm^−3^)	—	—	2 × 10^16^	1 × 10^19^	1 × 10^20^

While modelling the layers, defect layers are added to obtain more realistic results which are closer to experimental studies. For the perovskite absorber layer, the defect density (Nt) is taken as 1 × 10^14^ cm^−3^.

Defect type for the CTLs is kept as neutral. The type for energetic distribution is taken as single with defect (Nt) of 1 × 10^15^ cm^−3^. The Interfacial defects of HTL/absorber material and absorber material/ETL are also added between the layers. The simulation parameters of the materials are shown in table 1.

## 3. Results

### 3.1. IV characteristics

The IV characteristic curve are obtained for two carbon-based ETLs and two copper-based HTLs as shown in figure 2 while output performance are presented in table 2. All results are obtained by numerical modelling of PSC structures under STC. FTO/C_60_/CsGeI_3_/CuSCN/Au presented highest PCE of 12.24% with *J*_sc_ of 16.23 mA cm^−2^, *V*_oc_ of 0.87 V and fill factor (FF) of 85.86%.

**Table 2.** Output parameters of PSC structures.

structure	*V*_oc_ (V)	*J*_sc_ (mA cm^−2^)	FF (%)	efficiency (%)
PCBM/CsGeI_3_/CuSbS_2_	0.6962	19.452464	75.45	10.22
PCBM/CsGeI_3_/CuSCN	0.6951	19.478165	79.99	10.83
C_60_/CsGeI_3_/CuSbS_2_	0.8787	16.231885	82.93	11.83
C_60_/CsGeI_3_/CuSCN	0.8776	16.239076	85.86	12.24

**Figure 3.**
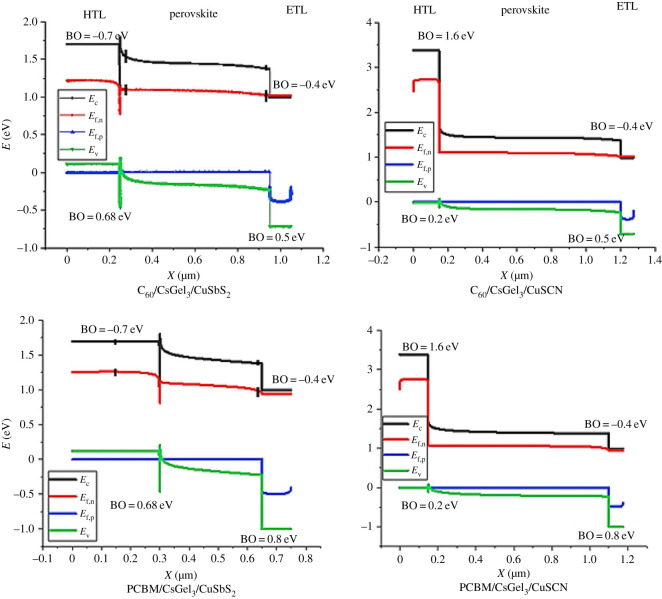
Energy band alignment of the PSC structures.

The difference in performance of the different PSC structures can be analysed from their energy band alignment, quantum efficiency, regeneration at interface and electric field at interface. Figure 3 shows the energy band alignment of the PSC structures while table 3 shows the valance band offset (VBO) and conduction band offset (CBO). The ideal band gap alignment of PSC should have minimum conduction band offset and maximum valance band offset of the ETL and perovskite material. This is to allow smooth flow of electrons from absorber to ETL and blocking of holes [18]. While the HTL and perovskite material should have minimum valance band offset and maximum conduction band offset. This is to allow the smooth flow of holes from absorber to HTL and blocking of electrons.

**Table 3.** VBO and CBO of the CTL with the perovskite.

CTL	CBO (eV)	VBO (eV)
CuSCN	1.6	0.2
CuSbS_2_	−0.7	0.68
PCBM	−0.4	0.8
C_60_	−0.4	0.5

Both of the carbon-based ETLs have almost identical band-gap alignment with perovskite material. Both have similar conduction band offset with the perovskite material of about 0.4 eV. This is because both materials have similar electron affinity. But the PCBM makes a larger valance band offset (0.8 eV) than C_60_ (0.5 eV) because of larger band gap. On the bases of band alignment of ETL and perovskite, both ETLs are able to block the holes and allow flow of electrons with small recombination owing to the CBO being slightly above 0.3 eV.

Variation in the performance is owing to the band alignment formed by the HTLs. CuSCN has perfect band alignment with minimum offset between the valance bands and maximum offsets between the conduction bands. The flow of holes is regulated from the absorber to the HTL smoothly with minimum recombination and blocks the electrons [19], while, on other hand, CuSbS_2_ has little band offsets at both the conduction and valance band alignment with the sharp vertical spikes at both interfaces. These sharp spikes at the interfaces cause trapping of charge carriers [20]. Holes are captured in these spikes and cause hurdles in the hole movement, which increases recombination rate and reduces performance.

### 3.2. Quantum efficiency

**Figure 4.**
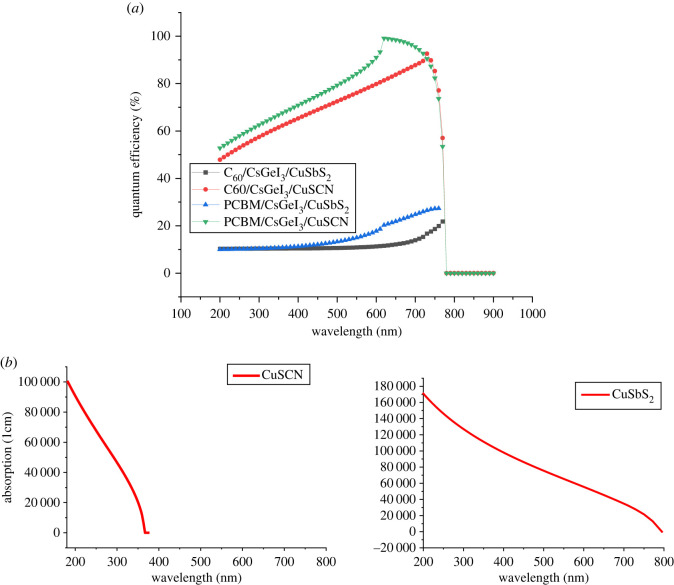
(*a*) Quantum efficiency of simulated structures. (*b*) Optical absorption of HTL material.

Quantum efficiency represents the photosensitivity of the device. It represents the ratio of potential incidents photons that are converted to electrons [10]. Quantum efficiency significantly depends on the band gap of the front material and the absorption of the absorber. Materials of larger band gap will potentially have higher quantum efficiency and vice versa [11]. This is because larger band-gap materials have low absorption and large transmittivity of the optical spectrum. This allows maximum light to reach the absorber where the charge carriers are produced. From figure 4*a*, it can be noted that CuSCN has higher quantum efficiency than CuSbS_2_ because it has higher band gap. CuSbS_2_ has lower band gap which causes the HTL to absorb more light spectrum than CuSCN, allowing less light to transmit to the absorber. This reduces the photo-generation concentration. This is further verified from the optical absorption graph of the two HTLs shown in figure 4*b*.

### 3.3. Electric field at interface

**Figure 5.**
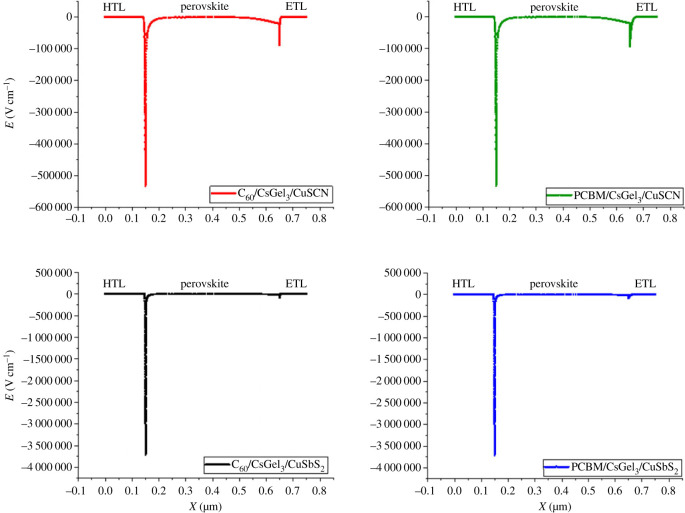
Electrical potential distribution.

The efficiency of the solar cell highly depends on electronic field at the interfaces [12]. The higher the potential field, the more charge carriers will be successfully separated and transported to the CTLs. From figure 5, it can be noted that both the ETL/perovskite and HTL/perovskite produce electric fields at interface. The HTL/perovskite electric field is much larger than the ETL/perovskite electric field. This means that the HTL increases the separation of charge carriers in the absorber. The CuSbS_2_ have significantly large electric field owing to the spikes at the interface in the band alignment. The spike produces higher built-in potential at the interface. But this does not increase the performance of the cell because the CuSbS_2_ has low conduction band offset with the perovskite. This leads to electrons also flowing to the HTL and causing recombination.

### 3.4. Recombination of charge carriers

**Figure 6.**
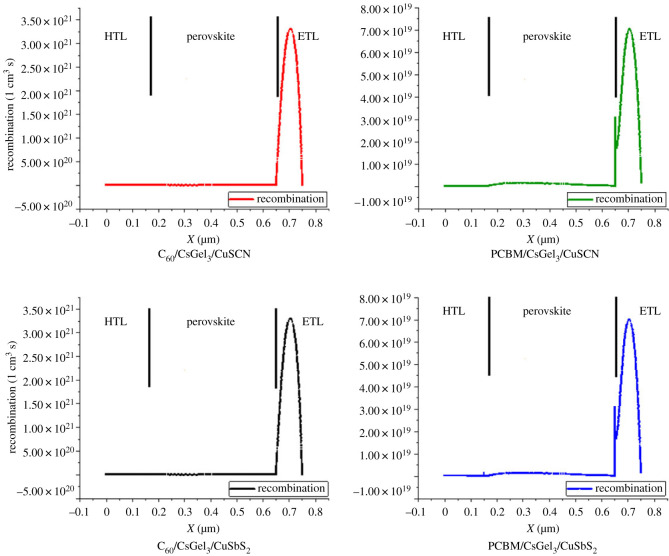
Recombination of charge carriers.

Recombination occurs when electrons are not collected and recombine with holes again. From figure 6, it is to be noted that recombination occurs at both interfaces. But there is high rate of recombination at ELT side. This is because of the conduction band offset of the ETL with the perovskite being larger than 0.3 eV. Because of the higher band offset, all electrons are not able to pass through [21]. The higher offset blocks some electrons, which causes recombination. Recombination may also occur because of low quality of the film or interface defects [22]. The recombination at the interfaces can be reduced by using a passivation layer between the CTL and perovskite layer. The passivation layer aids in transferring the charge carriers between the two layers and reduces the recombination [23]. Another technique through which the recombination can be minimized is by reducing the valance and conduction band offset of the materials through electron affinity/band gap tuning. By minimizing the band offset, maximum charge carriers will successfully cross over to the CTL [24]. Reducing the crystal defects through careful controlled environment fabrication techniques has also been shown to significantly reduce the defect density of the cell, which leads to reduced recombination [25].

### 3.5. The impact of absorber material thickness on perovskite solar cell

The role of absorber material is of significant importance in the PSC. When the photons strike the absorber, charge carriers are generated. With the increase in thickness of the absorber material, the probability of absorbing photons of higher wavelengths also increases significantly, increasing the charge carriers produced, which in turn improves the PCE [26,27].

**Figure 7.**
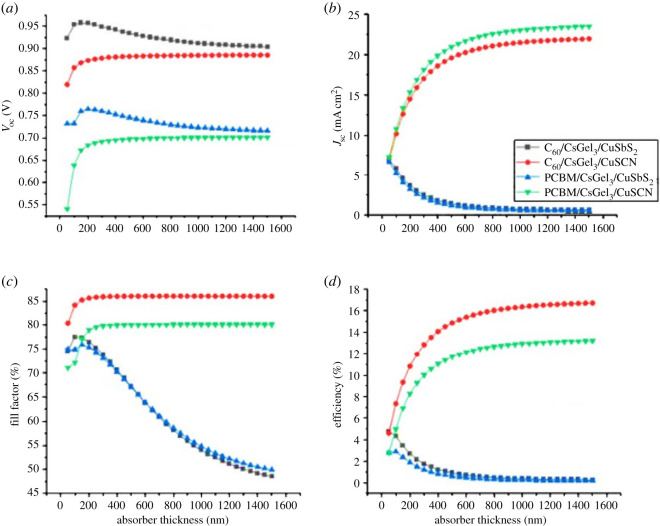
The impact of absorber material thickness on (*a*) *V*_oc_ (*b*) *J*_sc_ (*c*) FF (*d*) PCE.

In the discussed structures, absorber thickness is varied from 50 to 1500 nm with the increment of 50 nm to assess its effect on the performance and stability of PSCs with different HTL and ETL materials. The results in figure 7 show absorber material's thickness impact on different device parameters. Figure 7*a* shows that the *V*_oc_ of PSC slightly increases up to 150 nm and then becomes constant. High *V*_oc_ indicates high electron mobility [28]. From figure 7*b,d*, it can be concluded that the *J*_sc_ and PCE increase for CuSCN till saturation point owing to the increase in photo-generated ions. While for CuSbS_2_ the trend is the reverse, as it decreases with increase in absorber material thickness. This is because of the large traps produced at the interface of the structure as shown in the energy band alignment (figure 3). These traps capture the holes when they are being transferred from the absorber to the HTL. This increases the rate of recombination. The more the holes are captured, the faster the recombination rate and lower the PCE [29]. The decrease in PCE for CuSbS_2_ structures is also owing to increase in series resistance which increases the recombination of charge carriers [16,29].

The best results are obtained for C_60_/CsGeI_3_/CuSCN with *V*_oc_ of 0.8854 V, *J*_sc_ of 21.5687 mA cm^−2^, FF of 86.02% and PCE of 16.43%. The optimized parameters are mentioned in table 4. 

From the graphs in figure 7, it can be concluded that output parameters of the device are highly dependent of the absorber thickness. *J*_sc_ and PCE of the PSC increase with increase in absorber thickness till saturation point. With increase in thickness, more light spectrum is observed with higher wavelength. It also helps incident photons to produce more charge carriers [29]. Moreover, with further increase in thickness, the performance of the PSC degrades. It is because the rate of recombination increases.

### 3.6. The impact of charge transport material thickness

**Figure 8.**
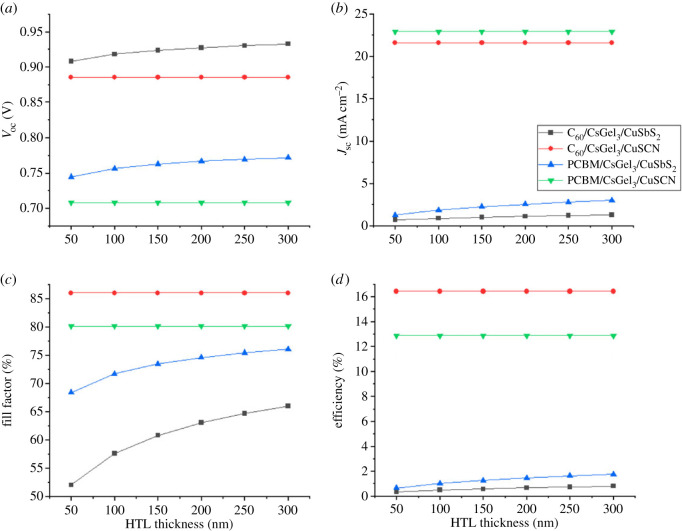
The impact of HTM thickness on (*a*) *V*_oc_ (*b*) *J*_sc_ (*c*) FF (*d*) PCE.

Once the thickness of absorber layer is optimized, HTM thickness is optimized next. It is incremented from 50 to 300 nm as shown in figure 8. The thickness of the other two layers (absorber and ETM) is kept unchanged. Once the optimized thickness for HTM is achieved, it is kept constant along with optimized absorber thickness to find the optimized thickness of the ETM layer. The same process of HTM is followed for ETM.

Both the CTLs have a very basic role in the structure of PSCs. Their function is to separate and transfer the photo-generated charged ions to the electrodes from absorber. The CTM also serves as a buffer barrier between them [30,31]. Increasing the CTM thickness results in increasing the cells series resistance. This makes it difficult for the charges to reach the electrodes. Hence, recombination occurs and reduces the PCE.

**Figure 9.**
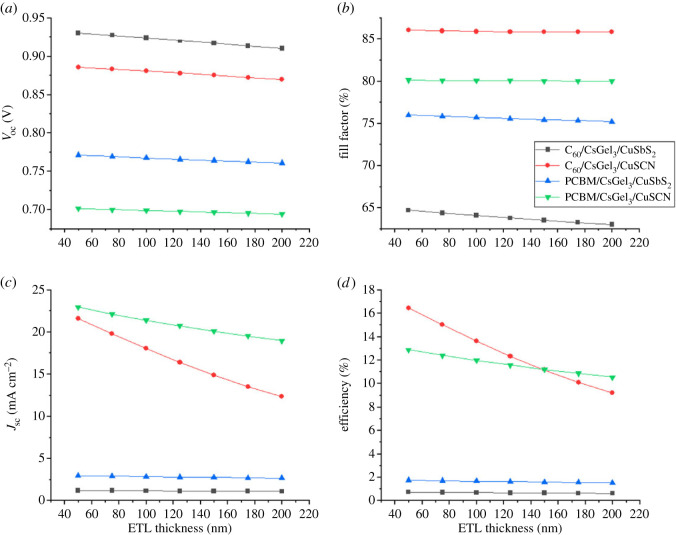
The impact of ETM thickness on (*a*) *V*_oc_ (*b*) FF (*c*) *J*_sc_ (*d*) PCE.

The results in figure 8 show that increasing the thickness of HTM has slight to no effect on the structure's performance. This is because the copper materials are highly conductive and the effect of series resistance is negligible. Similarly, the results in figure 9 show that increasing the thickness of both ETM reduces the PCE of the PSC because of the increase of the series resistance.

### 3.7. Absorber material doping optimization

**Figure 10.**
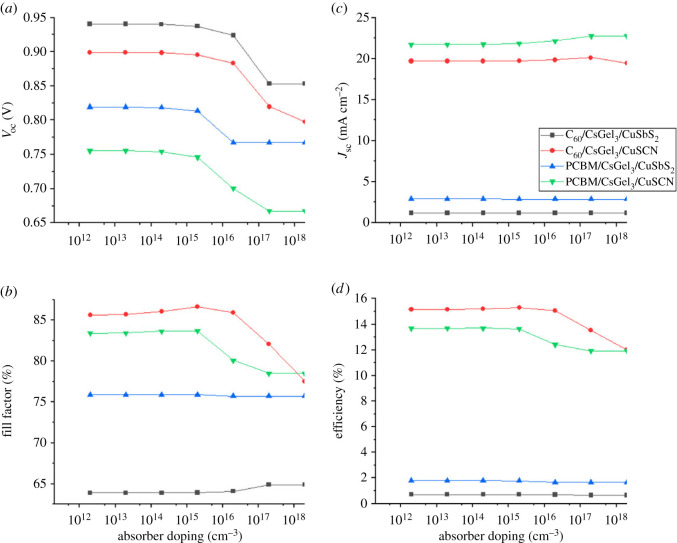
The impact of absorber doping on (*a*) *V*_oc_ (*b*) *J*_sc_ (*c*) FF (*d*) PCE.

To analyse the effect of varying absorber doping concentration on PSCs, the acceptor doping concentration (*N*_A_) is changed from 10^12^ to 10^18^ cm^−3^ as shown in figure 10.

The performance of the PSC may be improved by increasing the concentration of charge carriers through doping [32,33]. However, very high doping of the absorber layer may lead to decline in the device performance. This is because high doping produces traps in the absorber layer which affects the mobility of the charge carriers, leading to increase in recombination. Very high doping also affects the semi-conductor behaviour of PSC by making it more metallic in nature.

It is evident from the results in figure 10 that the PCE of the device is almost constant with very slight increase when the doping concentration is increased from 10^12^ to 10^15^ cm^−3^. However, further increasing the doping concentration reduces the PCE and *V*_oc_ for CuSCN structures while a slight increase is seen for CuSbS_2_ structures.

It can also be observed that *J*_sc_ almost remains constant and slightly increases in the case of CuSCN structures. While the FF has shown stable behaviour up to 10^15^ cm^−3^ but on further increase in *N_A_* the FF drastically decreases.

### 3.8. Charge transport material doping optimization

**Figure 11.**
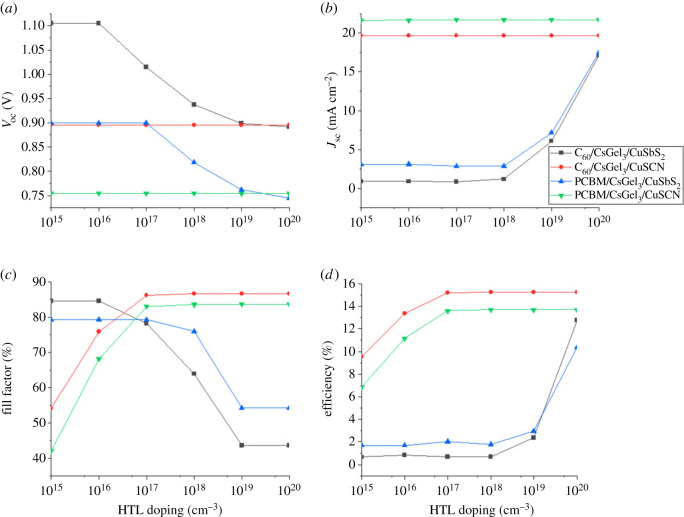
The impact of HTM doping concentration on (*a*) *V*_oc_ (*b*) *J*_sc_ (*c*) FF (*d*) PCE.

**Figure 12.**
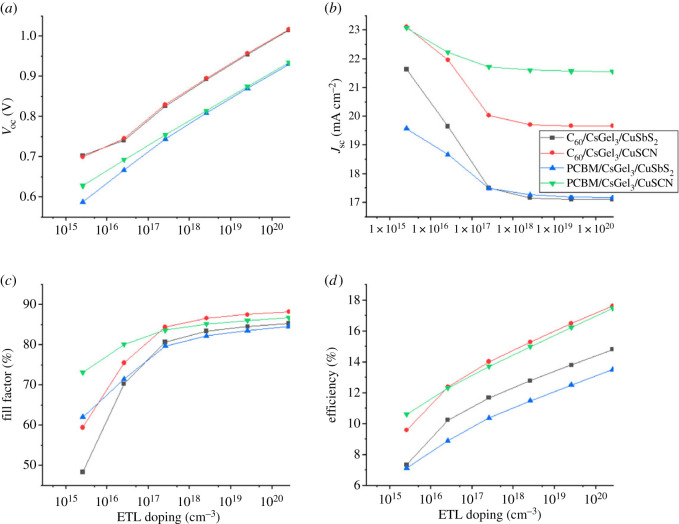
The impact of ETM doping concentration on (*a*) *V*_oc_ (*b*) *J*_sc_ (*c*) FF (*d*) PCE.

The doping concentration of CTM is changed from 10^15^ to 10^20^ cm^−3^ to study the impact of CTM doping concentration on PSC performance. For HTM the acceptor doping concentration (*N_A_*) is changed, whereas for ETM donor doping concentration (*N_D_*) is varied. The effect of HTM and ETM doping on PSC performance are shown in figures 11 and 12.

The task of CTM is to ensure that ions move smoothly from the absorber material to their respective electrodes. For the smoothness transition, the conductivity of the CTMs should be maximum while resistivity needs to be minimum. This can be achieved by the controlled doping of the CTMs [31,34]. The conductivity of the CTMs increases with the increase in doping concentration. It results in increasing the electrical field at the absorber interface which leads to increase in separating charge ions and increasing the PCE. At high doping, some materials see a decline in performance because of the Moss–Burstein effect.

From figure 11, it can be noted that *V*_oc_ is constant for CuSCN structures with slight increase in PCE. Variation in *J*_sc_ is almost negligible and PCE increases till saturation point where it becomes constant. While for CuSbS_2_ structures the decline in *V*_oc_ is because of increasing number of impurities (traps) owing to the doping. It also shows the incompatibility of CuSbS_2_ with the absorber material. The increase in *J*_sc_ and PCE for CuSbS_2_ at higher doping is because of generation of a very high number of charge carriers in it [29].

Similarly, from figure 12, it can be noted the *V*_oc_ firmly increases with increase in ETL doping for all structures. The electric potential increases with the increase in doping concentration because it pushes the e-h pairs to move freely from the traps. Which results in a significant increase of PCE and FF. The optimized doping for CuSCN HTL is 10^19^ cm^−3^, and 10^20^ cm^−3^ for CuSbS_2_. Whereas for ETL, the optimized doping is found to be 10^20^ cm^−3^.

### 3.9. Optimized perovskite PV cell

The optimized PSCs with their optimized thickness and doping concentration are shown in table 4. Different simulated structures and their results are compared in the table, and it can be observed that every structure has different optimized values of *V*_oc_, *J*_sc_, FF and PCE along with their thickness and doping values. The ETL doping values for all the cell structures are same.

**Table 4.** Optimized cell parameters with simulated output results of PSC.

cell structure	absorber thickness (nm)	ETL thickness (nm)	HTL thickness (nm)	absorber doping (cm^−3^)	HTL doping (cm^−3^)	*V*_OC_ (V)	*J*_SC_ (mA cm^−2^)	FF (%)	PCE (%)
C_60_/CsGeI_3_/CuSCN	1050	75	150	2 × 10^15^	1 × 10^19^	1.017	19.65	88.13	17.61
C_60_/CsGeI_3_/CuSbS_2_	700	100	250	2 × 10^15^	1 × 10^20^	1.015	17.1	85.28	14.79
PCBM/CsGeI_3_/CuSCN	950	75	150	2 × 10^14^	1 × 10^19^	0.934	21.55	86.78	17.47
PCBM/CsGeI_3_/CuSbS_2_	350	100	300	2 × 10^14^	1 × 10^20^	0.93	17.16	84.50	13.49

### 3.10. Absorber defect density

The defect density of the absorber material is a major parameter that affects the output of the PV cell. With the rise in defect density concentration of the absorber material, the chances of the recombination also increase. It degrades the stability of the PSC and the overall behaviour of the device. Recombination occurs owing to increase in trap levels. The diffusion length also reduced with increase in defect density owing to the traps reducing the carrier lifetime of the charge particles [35].

**Figure 13.**
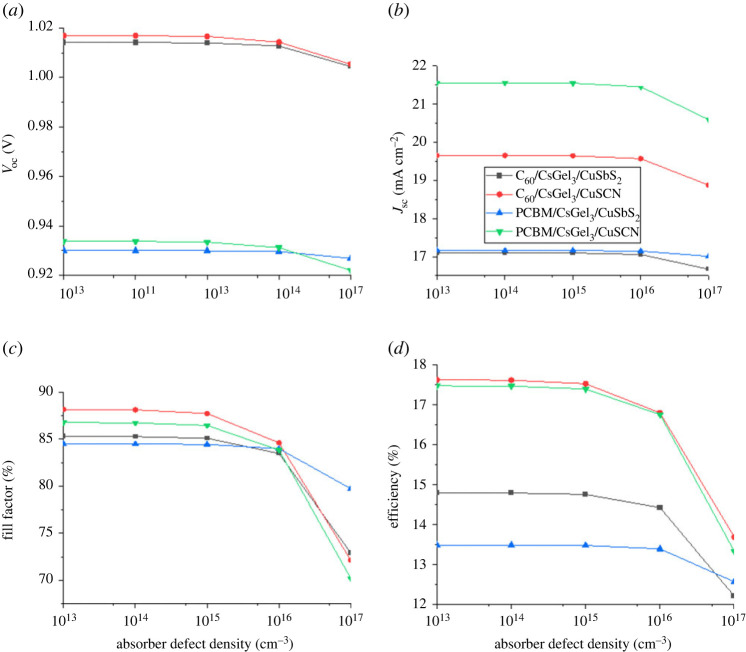
The impact of absorber defect density on (*a*) *V*_oc_ (*b*) *J*_sc_ (*c*) FF (*d*) PCE.

From figure 13, it can be noted that the absorber defect density has no effect on the PSC when it is increased up to 10^15^. However, when the Nt is further increased we see a drastic decrease in performance. This is because higher Nt leads to higher number of trap states in the absorber, which in turn increase recombination of charge carrier. This reduces the PCE of the PSC. The same trend is followed by *J*_sc_, *V*_oc_ and FF.

### 3.11. The impact of interface defects

Perovskite is a multi-layer device having a number of layers. These layers are fabricated over one another using different fabrication techniques. The interface defects between any two layers depend on the deposition technique of the layers. One of these interfaces is HTM/perovskite while the second is perovskite/ETM [36,37]. Increase in the interface defects causes a rise in the resistance of the cell and also raises the trap levels at the interface. Owing to this, the recombination increases and leads to reduction in cell performance [38]. The defects of both interfaces are changed from 10^10^ to 10^16^ cm^−3^ for each structure to look out for the effect, and the cell's output performance is studied.

**Figure 14.**
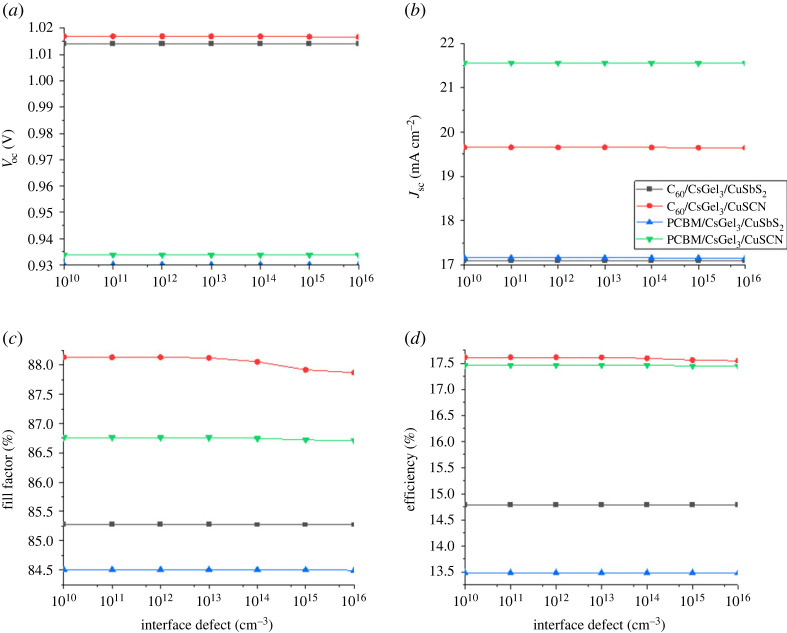
The impact of HTM/perovskite interface defects on (*a*) *V*_oc_ (*b*) *J*_sc_ (*c*) FF (*d*) PCE.

**Figure 15.**
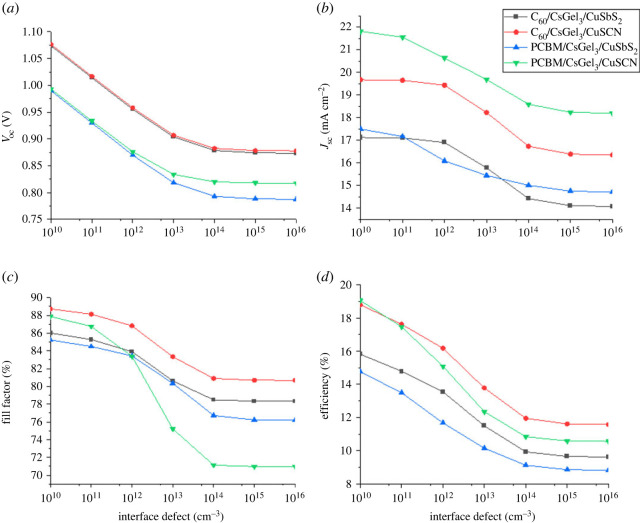
The impact of perovskite/ETM interface defects on (*a*) *V*_oc_ (*b*) *J*_sc_ (*c*) FF (*d*) PCE.

From figure 14, it can be concluded that HTM/perovskite interface defects have almost no effect on the output parameters of the device. This is owing to the higher conductive nature of copper-based HTM. While the results in figure 15 show that the PCE of all the structures reduce with the increase in perovskite/ETL defects. This is because the increase in number of trap levels increases the recombination at the interface. Similarly, the *J*_sc_ and PCE also decrease with an increase in Nt. Moreover, it is worth mentioning that if we want to increase the PCE and performance of the device we need to keep the interface defects as low as possible.

### 3.12. The impact of working temperature

**Figure 16.**
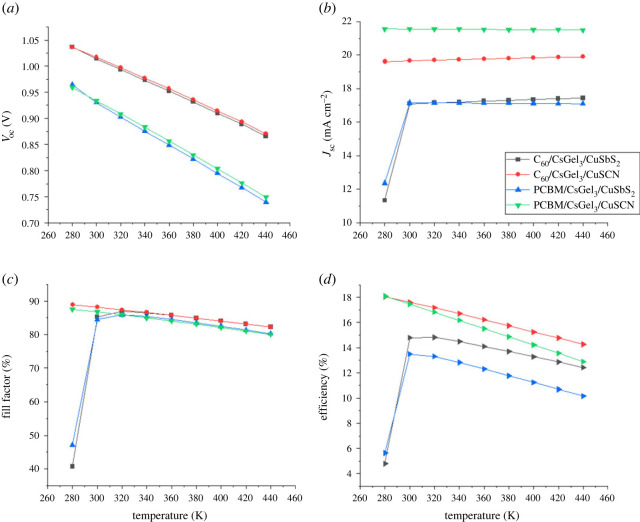
The impact of the working temperature on (*a*) *V*_oc_ (*b*) *J*_sc_ (*c*) FF (*d*) PCE.

The working temperature has a significant effect on the PSC's output performance. The majority of solar cells are highly stable at room temperature (300 K) and outperform at higher temperatures. To look into the effect of temperature on the device's performance and stability at high temperatures, the working temperature is changed from 280 K to 440 K with an increment of 20 K. Figure 16 shows the response of the solar cell as a parameter of temperature.

From the results of figure 16, it can be noted that the PCE and FF decrease with an increase in temperature. This is to the reverse saturation current's temperature dependency. Increase of temperature also leads to high recombination of ions, which causes the band gap to be unstable. *J*_sc_ remains constant while *V*_oc_ significantly reduces because of the absorber defects.

## 4. Conclusion

In this work, four PSC structures were numerically modelled using SCAPS-1D. CsGeI_3_ was used as the absorber layer, while two carbon-based ETLs and copper-based HTLs were used. The effect of varying thickness, doping, absorber defects, interface defects and temperature stability on the efficiency and the output performance of a PSC is thoroughly investigated. We can conclude from the final results that with the change in thickness of the absorber layer the PCE of the device increases till the saturation point, after which the output performance becomes constant for a while and then on the decline owing to the recombination of charge carriers. The CTM combination has great effect on the optimized thickness, with each structure giving a different value. The doping concentration had little effect on the performance of the cell when increased in the absorber layer, while it significantly improved the performance of the cell when increased in the CTM. From this study, it can also be concluded that Cu- and C-based CTM produce stable PSC. It was noted that the performance of PSC was highly degraded with the increase in absorber defects and interface defects. In the same way, with the increase in working temperature, the stability of the device decreased. The simulated results showed that for C_60_/CsGeI_3_/CuSCN, the open-circuit voltage (*V*_oc_) of 1.0169 V, short-circuit current density (*J*_sc_) of 19.653 mA cm^−2^, FF of 88.13% and the PCE of 17.61% were obtained.

Table 5 shows the comparison between the CsGeI_3_-based PSC results obtained from the present work and the results found in the literature. It has been noticed that Spiro-OMeTAD is used as the HTL in all other studies while different materials are used as ETL. Contrasting results have been achieved by their findings with PCE ranging from 5% to 17%. The non-optimized PSCs in the present work are also in line with their findings. However, by replacing the Spiro-OMeTAD with CuSCN as HTL and systematically optimizing each parameter of the structure, the **C_60_/CsGeI_3_/CuSCN** outperformed the previous ones.

**Table 5.** Comparative analysis of present work with literature.

structure	*V*_oc_ (V)	*J*_sc_ (mA cm^−2^)	FF (%)	efficiency (%)	reference
C_60_/CsGeI_3_/CuSCN	1.017	19.65	88.13	17.61	present work
TiO_2_/CsGeI_3_/Spiro-OMeTAD	1.04	23.31	75.46	18.30	[5]
C_60_/ CsGeI_3_/Spiro-OMeTAD	0.64	20.64	63.30	8.45	[15]
PCBM/CsGeI_3_/Spiro-OMeTAD	0.46	20.62	54.67	5.27	[15]
SnO_2_/CsGeI_3_/Spiro-OMeTAD	0.63	21.62	61.99	8.46	[15]

## Data Availability

The datasets generated and analysed during the current study can be accessed using the link: https://doi.org/10.5061/dryad.n5tb2rbzs [39].
